# Supplementary material for: Photoproducts of the Photodynamic Therapy Agent Verteporfin Identified via Laser Interfaced Mass Spectrometry
Source: Molecules. 2020 Nov 12;25(22):5280. doi: 10.3390/molecules25225280 (PMC7696214; doi:10.3390/molecules25225280)
Supplement: Supplementary file 1 [file molecules-25-05280-s001.pdf]

# Supplementary Material

# Photoproducts of the Photodynamic Therapy Agent Verteporfin Identified via Laser Interfaced Mass Spectrometry

Chris Furlan [1], Jacob Berenbeim[1], Caroline Dessent [1] *

Department of Chemistry, University of York, Heslington, York, YO10 5DD, UK

**Contents**

S1. CID of protonated verteporfin

S2. UV-Vis spectrum of verteporfin in acetonitrile solution

S3. Photolysis of verteporfin in acetonitrile solution

## Section S1: CID of protonated verteporfin

The mass spectrum of the fragments produced when CID is applied is shown in Figure S1.a. The protonated verteporfin (parent ion) is at m/z 719.3 while the other signals at lower m/z are the fragments detected. The CID plot (Figure S1.b) is obtained by dividing the intensity of each signal by the sum of the intensities of all signals and these values are plotted vs CID voltage. Increasing the CID voltage further doesn't change the relative production of the fragments (Plateau).

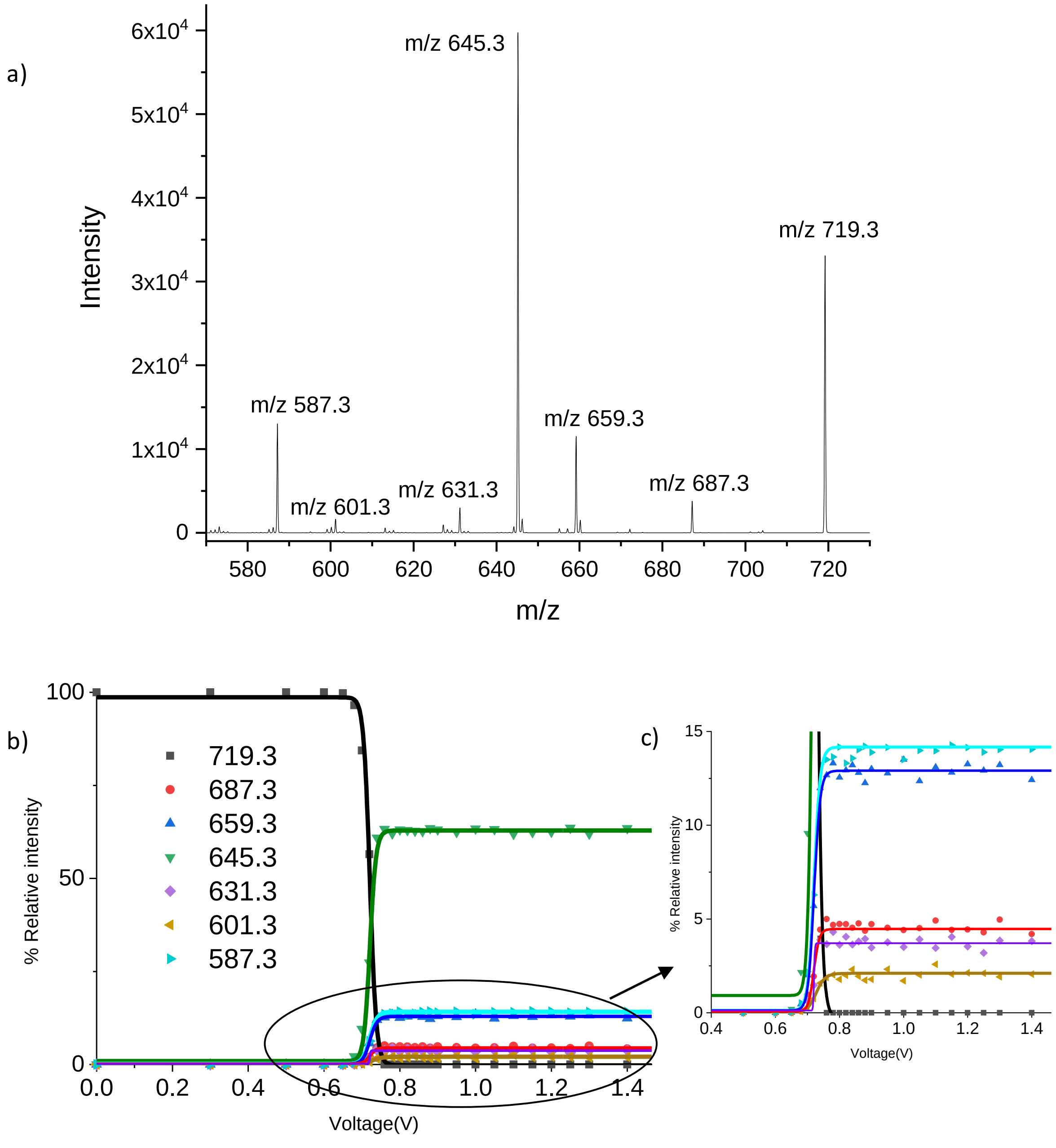


**Figure S1** CID of protonated verteporfin: a) MS spectra averaged across the whole CID voltage range. b) Plot of relative intensity against CID voltage. c) A vertically expanded view of the CID results to allow visualization of the minor fragments area of the plot.

## Section S2: UV-Vis spectrum of verteporfin in acetonitrile solution

Below the UV-Vis spectra of a verteporfin solution in MeCN is reported. The Soret band peaks at 413 nm and the Q band at 686 nm; this later is the band excited with red light during photodynamic therapy.

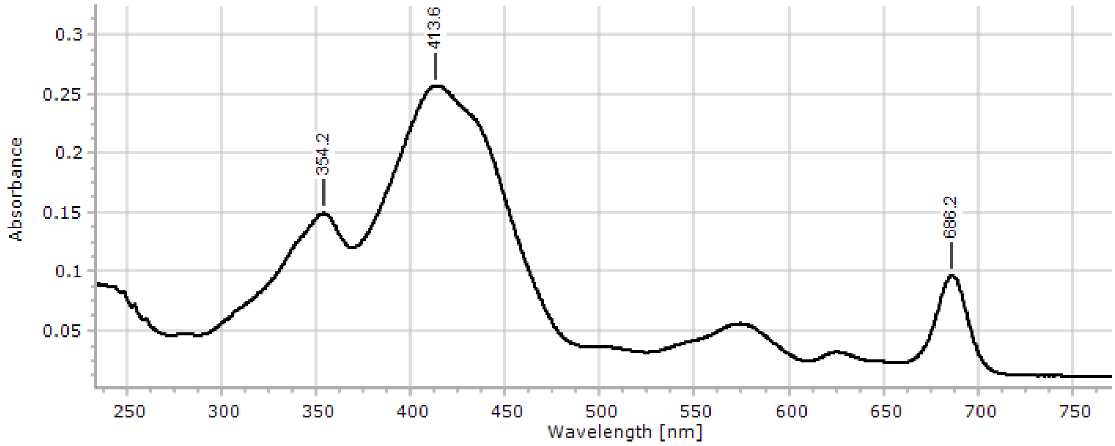


**Figure S2** UV-Vis spectra verteporfin in MeCN

## Section S2: Photolysis of verteporfin in acetonitrile solution

The solutions of verteporfin in MeCN become transparent after being exposed to light in the photolysis experiments. The solutions change color from pale yellow to transparent (Fig.S3) and no absorption peak can be detected on UV-Vis spectra across the 300-780 nm range (photobleaching).

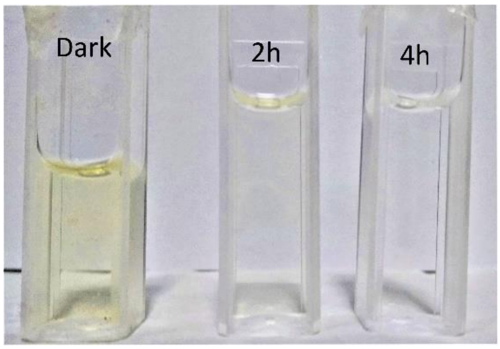


**Figure S3** Same concentration verteporfin solutions in MeCN without light exposure (dark), 2h and 4h inside the 365 nm photolysis cell. The color of the solution changes from pale yellow to transparent suggesting a photobleaching process with destruction of the macrocycle.
